# Structural Basis of CO_2_ Adsorption in a Flexible Metal-Organic Framework Material

**DOI:** 10.3390/nano9030354

**Published:** 2019-03-04

**Authors:** Andrew J. Allen, Winnie Wong-Ng, Eric Cockayne, Jeffrey T. Culp, Christopher Matranga

**Affiliations:** 1 Material Measurement Laboratory, National Institute of Standards and Technology (NIST), Gaithersburg, MD 20899-8520, USA; winnie.wong-ng@nist.gov (W.W.-N.); eric.cockayne@nist.gov (E.C.); 2 AECOM Corporation, Pittsburgh, PA 15236, USA; jeffrey.culp@contr.netl.doe.gov; 3 National Energy Technology Laboratory (NETL), US Department of Energy, Pittsburgh, PA 15236, USA; matranga@netl.doe.gov

**Keywords:** flexible metal-organic frameworks, gate-opening effects, dual gas flow sorption, supercritical CO_2_ adsorption, small-angle X-ray scattering, small-angle neutron scattering, X-ray diffraction, neutron diffraction, in situ operando studies, density functional theory

## Abstract

This paper reports on the structural basis of CO_2_ adsorption in a representative model of flexible metal-organic framework (MOF) material, Ni(1,2-bis(4-pyridyl)ethylene)[Ni(CN)_4_] (NiBpene or PICNIC-60). NiBpene exhibits a CO_2_ sorption isotherm with characteristic hysteresis and features on the desorption branch that can be associated with discrete structural changes. Various gas adsorption effects on the structure are demonstrated for CO_2_ with respect to N_2_, CH_4_ and H_2_ under static and flowing gas pressure conditions. For this complex material, a combination of crystal structure determination and density functional theory (DFT) is needed to make any real progress in explaining the observed structural transitions during adsorption/desorption. Possible enhancements of CO_2_ gas adsorption under supercritical pressure conditions are considered, together with the implications for future exploitation. In situ operando small-angle neutron and X-ray scattering, neutron diffraction and X-ray diffraction under relevant gas pressure and flow conditions are discussed with respect to previous studies, including ex situ, a priori single-crystal X-ray diffraction structure determination. The results show how this flexible MOF material responds structurally during CO_2_ adsorption; single or dual gas flow results for structural change remain similar to the static (Sieverts) adsorption case, and supercritical CO_2_ adsorption results in enhanced gas uptake. Insights are drawn for this representative flexible MOF with implications for future flexible MOF sorbent design.

## 1. Introduction

There is continued and sustained research interest in developing a better understanding of the properties of flexible porous coordination polymer (PCP) or flexible metal-organic framework (MOF) materials, especially those relevant to their selective gas adsorption capabilities [1,2,3,4,5]. Such an interest is based on the potential of PCPs or MOFs to address commercial needs for molecular sorption and sensing, enhanced gas recovery, carbon mitigation, and other gas storage applications [6,7,8,9,10]. While many MOFs are not found to change in structure significantly when guest solvent or gas molecules are adsorbed or desorbed, several flexible MOFs have been discovered that exhibit reversible structural transitions between nanoscale low-porosity and high-porosity states during the adsorption and desorption of gases [11,12,13,14,15]. Such structurally-dynamic flexible MOFs can show adsorption isotherms characterized by step-shape features where the pore system opens to accommodate the guest molecule. Below the step, little adsorption occurs, while the physical process at the step can be described as a “gate opening” event [16,17,18,19,20]. Another important aspect of the sorption isotherms is that the desorption branch frequently exhibits hysteresis with respect to the adsorption branch of the isotherm [21,22,23,24,25]. The temperature and pressure conditions that lead to gate opening can vary significantly for different guest molecules. In soft porous crystals, it is also possible to have behavior that is far more complex than a simple guest-induced gate opening such as “breathing” effects with multiple steps in the adsorption and/or desorption branches of the isotherm [26,27,28,29,30]. Meanwhile, the frequently observed hysteresis in the isotherm curve can be exploited as a basis for separating different gas species having different adsorption or desorption threshold pressures [31,32,33,34,35].

Understanding the structural dynamics that underlie the adsorption and desorption properties of flexible MOFs is a key requirement for improving and optimizing their performance to meet given selective gas sorption or separation requirements. Unfortunately, obtaining such insights frequently proves challenging. In part, this is due to there being no single structurally-dynamic, gate-opening mechanism ubiquitous across all flexible MOF materials [36]. More importantly, the flexible nature of the polymer linkers incorporated into the overall structure of flexible MOFs, results in some uncertainty and variability in any structure being determined. Thus, each flexible MOF, or class of flexible MOFs, must be investigated individually [37]. In this connection, since their original synthesis by Culp et al., [23], several of the present authors have studied members of the PICNIC (pillared cyanonickelate) class of flexible MOFs, which have a dynamic pillared cyanonickelate architecture [38,39]. This class of flexible MOFs incorporate metal ions bridged through organic linkers into extended coordinate-covalent networks [40]. This and similar architectures offer enormous diversity in the materials design of possible flexible MOFs, arising from an ability to alter the coordination geometry and organic linker properties in numerous combinations, thus creating porous networks of widely varied nanoscale size and chemical nature [41,42]. One of the more representative model PICNIC flexible MOFs, known to exhibit a high degree of CO_2_ adsorption and desorption in particular, is an effective extended 3D Hofmann clathrate analog: a pillared Ni(1,2-bis(4-pyridyl)ethylene)[Ni(CN)_4_], compound denoted as NiBpene [23].

NiBpene has been the subject of several studies by some of the present authors [23,39,40,43,44,45]. It has a characteristic CO_2_ adsorption/desorption isotherm exhibiting hysteresis between the adsorption and desorption branches and also features on the desorption branches that have been associated with structural changes, as measured by neutron diffraction (ND) and small-angle neutron scattering (SANS) [45]. Using single-crystal X-ray diffraction (XRD), it has been possible to achieve an a priori crystal structure determination, at least under conditions where ligands and solvents of crystallization, i.e., dimethyl sulfoxide (DMSO) and water molecules, occupy the nanoscale pores as a result of the crystallization technique used [44]. Consistent with the freely available crystallographic information files (CIF) uploaded in Reference [44], it was reported that when the cavities of the NiBpene structure (monoclinic *P2_1_/m*, with lattice parameters: *a* = 1.35941(12) nm, *b* = 1.43621(12) nm, *c* = 1.42561 nm, *β* = 96.141(2)°, unit cell volume: *V* = 2.7674(4) nm^3^, with standard deviation uncertainties in least significant digits given in parentheses), are full of dimethyl sulfonyl oxide (DMSO) solvent and extra ligands, the structure is wide open as a result of the near perpendicular geometry of the Bpene ligands (*β* ≈ 90°) relative to the basal 2-dimensional (2D) cyanonickelate net [23,39,44]. We envisage an analogous situation when the cavities are full of CO_2_, the volume of the unit cell would be the largest (*β* closest to 90°) as compared with the situation when CO_2_ is depleted. Conversely, when the structure ‘collapses’, the monoclinic *β* angle moves away from 90° and the Bpene linker *d*-spacing decreases. However, in reality, an absolute ‘guest-free’ situation might not occur with the material under ambient conditions due to the fact that a small number of guest molecules from the atmosphere could occupy the cavities. Nevertheless, one expects to see similar trends in the unit cell volume and *β* angle variation as a function of CO_2_ partial pressure, $p_{{\mathrm{CO}}_{2}}$. While the rapid in situ XRD measurements obtained here (see below) are not sufficient for full structure determinations, they are sufficient to follow the trends in the structural changes as a function of $p_{{\mathrm{CO}}_{2}}$ by performing least-squares lattice parameter refinements. Since lattice expansion (contraction) is expected as the temperature is increased (decreased) at a given pressure, some insights can also be gained by following lattice parameter changes as a function of temperature.

In summary, due to the variable configuration of the flexible pillared structure, broad peaks in the diffraction pattern can prove challenging for structural analysis across all of the ranges of adsorption/desorption conditions of interest [45]. Nevertheless, progress has been sufficient to enable density functional theory (DFT), which has been applied previously by some of the present authors to the case of CO_2_ adsorption in a 1D molecular sieve [46], to be applied to CO_2_ adsorption in NiBpene. Starting with the a priori single-crystal XRD structure previously determined, we here apply DFT (within significant uncertainties) to attempt predictions on how the NiBpene structure and its associated gas sorption properties change under realistic gas pressure conditions.

In this connection, we present new results for the adsorption of CO_2_ by NiBpene under dual gas conditions. The effects of dual gas adsorption as a function of CO_2_ partial pressure are illustrated for CO_2_/N_2_, CO_2_/CH_4_ and CO_2_/H_2_ static gas pressure conditions using SANS-based small-angle neutron diffraction. Structural changes associated with adsorption and desorption are further explored under in situ operando conditions for the same gas combinations under dual gas flow conditions. In this case, the measurements we present are based on combined rapid ultra-small-angle X-ray scattering (USAXS), small-angle X-ray scattering (SAXS) and powder XRD measurements [47,48,49], developed with others by one of the present authors (A.J.A), and both the adsorption and desorption processes can be followed. Applying the previously-determined structure results [44] together with the new powder XRD results, we now present DFT calculations that provide insights regarding CO_2_ sorption, in particular, under both single-gas and dual-gas sorption conditions. In particular, the DFT calculations show how the gas sorption properties can be associated with inferred changes in the NiBpene pillared structure, including the reversible 90° rotation of individual Bpene ligands. Finally, we also explore the partially-reversible enhanced CO_2_ adsorption by NiBpene observed to occur in the supercritical CO_2_ pressure regime. We consider how supercritical conditions enhance the CO_2_ adsorption in NiBpene, bringing the CO_2_ molecules per mole close to the theoretical maximum limit of five CO_2_ molecules per formula unit, predicted by the DFT calculations.

While the flexible nature of the NiBpene microstructure makes XRD determination of the structure under all adsorption conditions extremely challenging, just as for most flexible MOFs, it remains important to characterize gas sorption behavior and relate this to structure, as we illustrate below. We note how model calculations based on DFT simulations, such as those we present, can indicate how the microstructural design may be modified or how species can be added to improve gas sorption performance. Exciting opportunities exist to design new flexible MOF materials with enhanced gas sorption performance [50,51]. However, as we conclude, this requires that the crystal structure is sufficiently well characterized to provide all needed information for DFT to predict adsorption and desorption behavior.

## 2. Materials and Methods

### 2.1. Synthesis and Preparation of NiBpene Powder

All chemicals were purchased from Sigma-Aldrich (St. Louis, MO, USA) and used as received. The synthesis of polycrystalline NiBpene, comprising of Ni(Bpene)[Ni(CN)_4_] where the Bpene ligand has a composition: C_16_H_10_N_6_Ni_2_, followed a previously reported procedure [44]. In brief, a flask was charged with 4 mmol of polymeric Ni[Ni(CN)_4_]*_n_* hydrate. Using sequential incremented additions of 15 mL H_2_O, 15 mL concentrated ammonium hydroxide solution (≈ 28% by volume) and 15 mL of dimethyl sulfoxide (DMSO), the solid was dissolved slowly at room temperature while stirring. (Note: concentrated NH_4_OH must not be mixed directly with DMSO directly, or rapid outgassing of NH_3_ can occur). Once dissolved, 5.6 mmol of 1,2-bis(4-pyridyl)ethylene dissolved in 35 mL DMSO was added to the clear solution. The pressure in the flask was slowly decreased using a water aspirator to outgas the NH_3_ at a controlled and approximately constant rate, which was sufficiently slow to prevent excessive bubbling. After ≈ 90 min, the precipitated solid was filtered off and washed with a mixture of 1-part H_2_O, 3-parts DMSO, followed by acetone. The solid was then extracted in 100 mL of acetone at reflux for 2 h and isolated by filtration. In this process, 247 mg or 1.4 mmol of Bpene ligand was recovered on evaporation of the acetone filtrate. The extracted solid was then further refined in 50 mL toluene at reflux for 30 min to give a toluene-loaded sample free of Bpene guests as determined by thermogravimetric analysis (TGA). To facilitate sample activation for gas sorption measurements, toluene was replaced by acetone by extraction of the former in boiling acetone until TGA analysis showed complete exchange of toluene with acetone (after 1 h to 2 h). The adsorbed acetone was removed overnight at 90 °C under vacuum to yield 1.22 g (3.0 mmol) of guest-free Ni(Bpene)Ni(CN)_4_. This formula mass was verified from the residual mass of NiO after TGA in air to 550 °C.

### 2.2. Small-Angle Neutron Scattering and Diffraction under Static Dual Gas Conditions

SANS measurements were carried out at the NIST Center for Neutron Research (NCNR) using the NCNR’s two 30-m SANS instruments [52] with similar measurement configurations to those reported previously [45]. SANS measurements are used both to determine the scattering intensity profile, *I*(*q*), as a function of the magnitude of the scattering vector, *q*, where *q* = (4π/*λ*) × sin(*θ*), and *θ* is one half of the scattering angle and *λ* is the neutron wavelength, and to determine the position and intensity of the first small-angle diffraction peak, *q*_PK_, which is associated with the flexible MOF *d*-spacing (where *d* = 2π/*q*_PK_) most sensitive to the presence of adsorbed guest molecules. For all SANS measurements, λ = 0.51 nm. Using up to three instrument configurations, SANS data were obtained in the *q* range: (0.04 < *q* < 6) nm^−1^. This is sufficient to characterize both the powder morphology present and the internal nanoscale structure. The powder morphology determined from SANS has been reported previously [45], so only the data in the (3 < *q* < 6) nm^−1^ range has been used to provide the relevant neutron-based small-angle diffraction results reported here. In all SANS measurements, data were recorded on a two-dimensional detector. Using the NCNR SANS analysis software package developed using Igor Pro (Wavemetrics, Lake Oswega, OR, USA) [53], data were corrected for detector sensitivity, electronic and parasitic background effects, and sample absorption, and then calibrated against the incident beam flux and normalized to unit sample volume. Finally, the data were circularly averaged to obtain absolute scattered intensity *I*(*q*) versus *q* data.

In situ data, specifically for the diffraction peak associated with the main Bpene ligand *d*-spacing, were collected for activated NiBpene under vacuum, and in separate experiments at up to 17 bar of the following pure gases: CO_2_, N_2_, CH_4_, and H_2_, and also in 50/50 mixtures of N_2_, CH_4_ and H_2_ with CO_2_ at total pressures up to 34 bar. All measurements were carried out at 30 °C. Note that, with the relatively small amount of the NiBpene sample in the chamber (≈0.11 g), the large headspace volume (≈2 L) and consequent large number of moles of CO_2_ at 17 bar (1.35 mol CO_2_), changes in headspace composition of the gas mixtures due to any selective CO_2_ adsorption were negligible (<0.02%). The objective of these measurements was to follow the behavior of the main NiBpene ligand space diffraction peak as a function of CO_2_ partial pressure during dual gas adsorption and compare this both with the previously determined CO_2_ sorption isotherm for NiBpene and with the previously measured response of this peak during pure CO_2_ adsorption. Details of the isotherm measurement and the detailed setup for the in situ SANS experiments have been described previously [45].

### 2.3. Small-Angle X-Ray Scattering and Diffraction under Dual Gas Flow Conditions

The X-ray measurements reported here were conducted at the USAXS facility at the Advanced Photon Source (APS), Argonne National laboratory, Argonne, IL [47,48,49]. This facility provides combined USAXS, SAXS and wide-angle X-ray scattering (WAXS) measurements without disturbing the sample position within ≈6 min. In principle, these measurements cover a contiguous *q* range from 0.001 nm^−1^ to 60 nm^−1^. Here, the maximum *q* was limited to ≈ 45 nm^−1^ due to restrictions on the scattered beam path arising from the sample cell geometry. The USAXS instrument exploits Bonse-Hart-type double-crystal optics and extends the SAXS *q* range down to 0.001 nm^−1^. For USAXS measurements, the beam size was 0.6 mm × 0.6 mm. To provide better signal-to-noise at high *q*, the USAXS instrument was supplemented with a Pilatus 100K detector (Dectris Ltd., Baden, Switzerland) in a conventional pinhole SAXS geometry. The SAXS *q* values were calibrated using an AgBe calibration standard. A beam size of 0.6 mm horizontal × 0.2 mm vertical was used for the SAXS measurements. The combined accessible *q* range for USAXS and SAXS is 0.001 nm^−1^ to 15 nm^−1^, and the combined dynamic range in linear intensity response exceeds 10 orders of magnitude. To evaluate changes in the atomic structure of NiBpene during gas adsorption and desorption, a modified Dectris Pilatus 300 KW detector was used to perform area-detector-based WAXS measurements providing XRD data in a *q* range from 14 nm^−1^ to the maximum accessible 45 nm^−1^. A NIST Standard Reference Material, SRM 660a (LaB6: lanthanum hexaboride) [54] was used to calibrate the *q* values and sample-to-detector geometry. The beam size used for WAXS measurements was the same as for the pinhole SAXS. The USAXS, SAXS and WAXS data were reduced and calibrated using the APS USAXS facility analysis software packages (especially Irena and Nika) [55,56] and developed using Igor Pro.

X-ray energies used for the USAXS, SAXS and WAXS measurements were 18 keV and 21 keV (*λ* = 0.06889 nm and 0.05904 nm, respectively). The use of this relatively high X-ray energy, together with a rapid overall measurement time, permits sample environments with substantial sample cell windows for studies requiring elevated pressures or temperatures. Here, a small parallel-sided powder sample (thickness ≈ 0.05 mm) of NiBpene was encapsulated in plastic tape, which was perforated to allow the inflow and outflow of gas. This sample was then mounted inside a pressure cell with polyamide film windows and the cell connected to an automated Hiden XCS dual gas flow system (Hiden Isochema Ltd., Warrington, UK). This system employs gas flow through the sample (at ≈100 mL/min) and a gate valve system to apply total gas pressures up to 50 bar. After initial activation of the sample by CO_2_ pressurizing to 5 bar followed by partial evacuation to ≈0.3 bar, USAXS/SAXS/WAXS measurements were then conducted as a function of gas pressure in separate experiments using pure CO_2_, N_2_, CH_4_, and H_2_ up to 18 bar, each followed by similar measurements with a dual gas flow of CO_2_/N_2_, CO_2_/CH_4_, and CO_2_/H_2_, respectively, up to a total pressure of 35 bar. In each case, the nominal 100 mL/min total gas flow was distributed 50/50 between the two gases, with corrections for the different gas constants applied automatically by the Hiden XCS control software. Thus, the USAXS/SAXS/WAXS measurements approximately followed the pressure conditions of the previously described SANS measurements except that the 50/50 partial pressures of the gas mixtures were achieved by dual gas flow control rather than use of a Sieverts gas-cart arrangement. In these studies, both the internal powder morphology and the main Bpene ligand spacing diffraction peak could be followed as a function of gas pressure conditions (as for SANS), but also the XRD pattern as a whole could be followed under the same conditions. The measurement configuration is shown schematically in the Supporting Information Figure S1.

### 2.4. Small-Angle X-Ray Scattering and Diffraction under Supercritical CO_2_ Conditions

Using a different sample environment configuration, USAXS/SAXS/WAXS measurements were also made of NiBpene under supercritical CO_2_ pressure conditions. In this configuration, the powdered sample was mounted in the center of a 1.5-mm quartz capillary plugged at both ends with porous glass wool. The capillary was connected to a Teledyne Isco 1000D syringe pump gas system (Teledyne, Lincoln, NE, USA) that enabled CO_2_ pressures to be increased into the supercritical regime. The measurement configuration is shown schematically in the Supporting Information Figure S2. This arrangement also incorporates a thermocouple mounted through one end of the capillary and heating coils to both calibrate and select the desired sample temperature. The sample was activated by a gentle purge of the system with flowing CO_2_, pressurizing the system to 5 bar of CO_2_ under static (non-flow) conditions, followed by a second gentle purge of flowing CO_2_, then the system was sealed and pressurized to the desired CO_2_ pressure under static non-flow conditions. Using this arrangement, USAXS/SAXS/WAXS measurements were carried out at several sub-critical CO_2_ pressure values with the sample held at 90 °C. Then, the system pressure was incremented in small steps through the supercritical gas transition at ≈73 bars CO_2_ to ≈80 bars CO_2_ gas pressure. Several pressures and temperatures were investigated in the supercritical CO_2_ regime, until finally the pressure was reduced in increments to ambient with the sample temperature at 60 °C. The USAXS/SAXS/WAXS measurement configurations were identical to those above used for the subcritical dual gas flow studies, except that the WAXS data *q* range was no longer constrained by the pressure cell design, so XRD data were obtained at the maximum measurable *q* of 60 nm^−1^.

### 2.5. Estimation of Lattice Parameter Changes in Response to Gas Adsorption/Desorption in NiBpene

The goal of lattice parameter estimation was to elucidate the structural flexibility of NiBpene as reflected by changes in the monoclinic unit cell *β* angle through determining trends in the response of the unit cell parameters as a whole, as a function of $p_{{\mathrm{CO}}_{2}}$ (or other gas partial pressure). The Jade Software Suite (Materials Data Inc., Livermore, CA, USA) was used for the least-squares refinements. Starting from the previously-published single-crystal structure of NiBpene [44] and DFT-predicted (see below) different-but-related cell parameters (monoclinic, *a* = 1.3784 nm, *b* = 1.4822 nm, *c* = 1.3818 nm, *β* = 108°), we performed least-squares refinements using the XRD peaks in the SAXS and WAXS regimes of eight selected experimental USAXS/SAXS/WAXS datasets. These XRD peaks cover a *q*-range from 0.028 nm^−1^ to 0.47 nm^−1^ (equivalent Cu*K*α2*θ* range from 4° to 70°). To determine the trend in unit cell volume and *β* angle in this series of XRD patterns, we also included (for room temperature data) powder diffraction data collected previously using a computer-controlled Philips powder diffractometer (Malvern Panalytical Inc., Westborough, MA, USA) with a Cu*K*α2*θ* range from 3° to 60°. However, most peaks were broad and weak for 2*θ* > 40°.

The XRD peaks for NiBpene are broad in general, and some are very weak. Particularly at higher diffraction angles, the peaks in rapid in situ USAXS/SAXS/WAXS datasets are also distorted in shape. It is difficult to assign a space group to these patterns, and therefore, they were refined by using the primitive lattice *P*. We did not use the space group *P2*_1_/m that was determined for the NiBpene single-crystal measurements [44], directly, because the crystal had different guests present, as well as extra ligands within the cavities. These could give rise to a different space group.

### 2.6. DFT Model Interpretation

**Figure 1:**
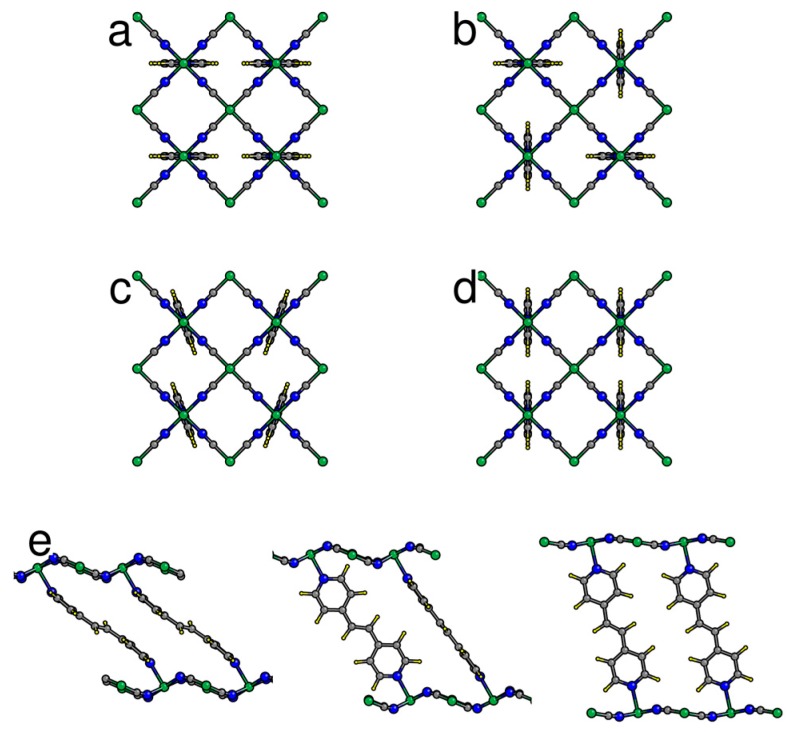
Examples of possible ordering patterns for Bpene pillars in NiBpene as projected onto the *bc* plane: (**a**) [b]-type: projected Bpene coordinates extend primarily along the *b* axis; (**b**) [bc]-type: an alternation of projected Bpene orientations along the *b* and *c* axes; (**c**) canted [c] type; (**d**) [c]-type; (**e**) schematic of NiBpene cell expansion upon CO_2_ sorption, projected onto the *ca* plane (CO_2_ not shown). Changes in Bpene orientation upon expansion are based on DFT calculations.

Density functional theory calculations were performed to complement the experimental measurements and to model the energetics and structural changes upon CO_2_ sorption in NiBpene. Although all current density functionals use some approximation to the unknown exact exchange-correlation functional, in practice, DFT that combines the Perdew–Burke–Ernzerhof (PBE) or solid-state PBE (PBEsol) generalized gradient approximation [57,58] with “Hubbard U” corrections for transition metal ions [59] and empirical van der Waals forces has been found to provide a good combination of speed and accuracy for the study of CO_2_ sorption in MOFs [60]. Flexible MOFs can have one or more elastic parameters that are extremely small [61]. Because of this, the Pulay stress (which normally causes only negligible strain error at the cutoff energy that we use) causes significant enough strain errors in NiBpene to make typical iterative full relaxation inefficient. We therefore use an “equation of state” approach to fully relax the structures where the total energy is calculated as a function of volume through a series of fixed volume (cell shape allowed to change) calculations [62]. The ground state volume is found via cubic spline interpolation and the ground state structure via fixed-volume relaxation at this volume. For NiBpene, tests on nickel oxide and planar metallocyanide compounds showed that a Hubbard value of U = 5.0 eV for Ni and empirical van der Waals forces treated using the Tkatchenko–Scheffler method with iterative Hirshfeld partitioning [63,64,65] reproduces the experimental structures well. These parameters were thus used for NiBpene. The Ni ions were fixed at the spin states expected for their local environments: high spin for the Ni coordinated by 6 N in empty cells and low spin for the Ni coordinated by 4 C. For all computations, a supercell of the primitive structure was taken with the lattice parameters *b* and *c* and doubled, allowing possible Bpene ordering superstructures to be investigated; see Figure 1. The spins for the high spin (hs) Ni ions were arbitrarily set to be antiferromagnetic for the nearest hs neighbors; in any case, spin interactions at 0.7 nm distance are expected to be very small. All possible orderings of the Bpene molecules along the crystal axes were investigated with *P2/c* monoclinic or higher symmetry, plus some special structures with lower symmetry. All relaxations were carried out at fixed volumes (cell shape allowed to relax) that were multiples of 0.025 nm^3^. The optimum cell volume was found via cubic spline interpolation of the results. The DFT calculations were carried out using the VASP software (VASP Version 5.4.4., VASP Software GmbH, Vienna, Austria) [66,67].

The lowest-energy configurations of each ordering type found for empty Ni-Bpene were used for CO_2_ studies. One CO_2_ per primitive cell at a time (four per supercell) was introduced into each structure; the lowest energy position and orientation for this CO_2_, and the relaxed structure, were found (as before, from a cubic spline fit to a series of fixed volume relaxations). Then, an additional CO_2_ per primitive cell was introduced and the optimization procedure repeated for this CO_2_. In each case, hundreds of initial combinations of positions and orientations were considered for the new CO_2_. We are thus confident that we have identified important low-energy configurations of CO_2_ molecules in NiBpene, but it is possible that collective rearrangements of the CO_2_ could lead to even lower-energy configurations. The calculations used *P2/c* monoclinic symmetry to match the experimentally known lattice type while avoiding the overlap of symmetry-equivalent CO_2_ molecules. Preliminary results for H_2_, N_2_ and CH_4_ sorption were similarly obtained for one fixed representative cell.

## 3. Results and Discussion

### 3.1. Dual Gas Adsorption under Static Pressure Conditions

**Figure 2:**
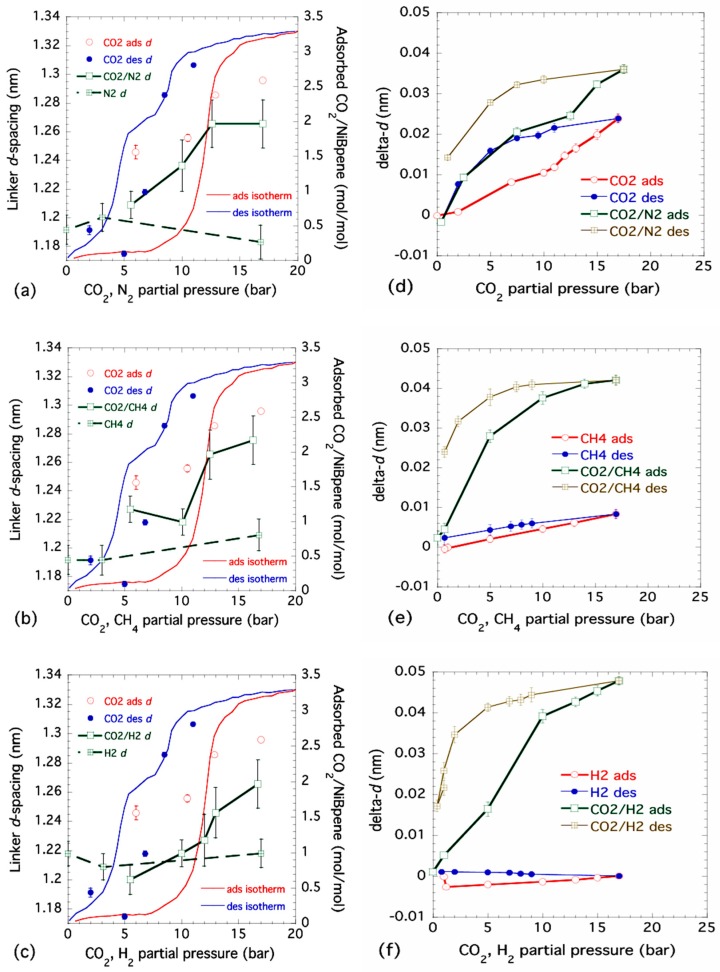
Variations in Bpene linker *d*-spacing with either pressure (pure gas) or partial pressure (dual gas). (**a**) N_2_ and CO_2_/N_2_, (**b**) CH_4_ and CO_2_/CH_4_, and (**c**) H_2_ and CO_2_/H_2_, show ND-based results under static gas pressure conditions, superimposed on measured CO_2_ adsorption/desorption isotherms measured at 30 °C. These plots show changes in *d*-spacing for pure CO_2_ adsorption (CO_2_ ads *d*) and desorption (CO_2_ des *d*). Additional points and lines refer to either pure gases (indicated) or dual gas combinations with CO_2_ (adsorption only). (**d**) CO_2_ and CO_2_/N_2_, (**e**) CH_4_ and CO_2_/CH_4_, and (**f**) H_2_ and CO_2_/H_2_ show XRD-based results for changes in Bpene linker *d*-spacing under gas flow conditions at ≈30 °C. These plots show changes in *d*-spacing for both adsorption (ads) and desorption (des) for pure gases and also dual gas combinations. In (**a**) to (**f**), vertical bars are computed standard deviation uncertainties based on Gaussian (ND) or Lorentzian (XRD) peak fits to the linker *d*-spacing peak.

SANS (low-angle diffraction) data were used to track variations in the main Bpene linker *d*-spacing as a function of pressure. The small-angle diffraction peak data are shown in the Supporting Information Figure S3, while the variations in linker *d*-spacing are shown superimposed on the previously measured isotherm curves for NiBpene in Figure 2 [45]. For pure CO_2_ adsorption, the linker *d*-spacing shows a rough correlation with the adsorption and desorption branches of the isotherm (indicated in Figure 2a–c). We associate this with the Bpene ligand pillars standing up and letting CO_2_ molecules adsorb into the caged cell structure. In the case of pure N_2_, CH_4_ or H_2_, Figure 2a–c, respectively, they showed little or no significant change in the linker *d*-spacing. This suggests that, under Sieverts-type gas dosing conditions at near ambient temperatures, each of these gases alone is not significantly adsorbed into the NiBpene structure over the experimental timescales of 15 min to ≈2 h. For the case of Sieverts-type dual gas dosing, where each gas is initially stored with CO_2_ to an equal partial pressure within the gas reservoir, variations in the linker *d*-spacing broadly follow the same trend as that for the pure CO_2_ case over the equivalent CO_2_ partial pressure range. However, the absolute increases in linker *d*-spacing are significantly less than in the pure CO_2_ case. Note that only changes during adsorption were followed, since changes in the ratio of the two residual gas partial pressures during desorption can result from any selective desorption of one gas ahead of the other. These observations suggest that while NiBpene may have some preference for CO_2_ adsorption under Sieverts gas dosing conditions on timescales of less than a few hours, complex cooperative gate-opening processes may occur with more than one gas present [38,68].

### 3.2. Dual Gas Adsorption and Desorption under Flow Pressure Conditions

Combined USAXS, SAXS and WAXS results are presented (for H_2_) in the Supporting Information Figure S4 and (for linker *d*-spacings) in Figure 2d–f for the following single-gas and dual-gas combinations under controlled flow conditions: CO_2_ and CO_2_/N_2_, CH_4_ and CO_2_/CH_4_ and H_2_ and CO_2_/H_2_. Changes in the microstructure and structure were followed as a function of both adsorption and desorption, because any selectivity in gas release from the sample that might occur during desorption does not affect the gas partial pressures under the dual gas flow conditions used.

In Figure 2d–f, the changes in Bpene linker *d*-spacing, delta-*d*, with respect to that measured at a pressure below 1 bar are plotted versus pressure. Note that for single gas flow, the plots are with respect to total pressure on the sample in the gas flow system. For the 50/50 dual gas measurements, the total pressure is double that shown but the gas-flow conditions enforce a 50% partial pressure for each component gas. This is achieved through an automated correction in the mass flow controller setting for each gas, according to the gas law specific to that gas. Also, plotting the changes in *d*-spacing here removes the effects of any deviations in the absolute *d*-spacing due to sample preparation or history. A comparison of Figure 2d with Figure 2a reveals that the effects of pure CO_2_ adsorption under flow conditions follow the same trends as under Sieverts gas dosing conditions, although the overall change in *d*-spacing during flow: (0.024 ± 0.001) nm, is somewhat less than in the Sieverts case: (0.050 ± 0.005) nm on adsorption and as much as (0.115 ± 0.005) nm on desorption. The effects of pure N_2_ sorption were not measured under flow conditions, but we note that the linker *d*-spacing variation for CO_2_/N_2_ dual gas flow, carried out immediately after the pure CO_2_ cycle, is enhanced significantly to (0.036 ± 0.001) nm, although following the same trend as for the effects of pure CO_2_ gas flow. In Figure 2e, the effects of pure CH_4_ flow on the *d*-spacing during adsorption and desorption confirm the hinted increase in *d*-spacing, (0.017 ± 0.015) nm, seen in the Sieverts-type static gas case shown in Figure 2b, with a reduced but less uncertain increase in *d*-spacing on adsorption under gas flow conditions of (0.008 ± 0.001) nm. However, there is a clear amplification in the variation in linker *d*-spacing in the case of dual CO_2_/CH_4_ gas flow: (0.042 ± 0.002) nm, compared to the case for pure CO_2_ flow versus total CO_2_ pressure. These observations are even stronger for the case of CO_2_/H_2_ dual gas flow (Figure 2f), where there is no significant effect due to H_2_ adsorption alone (just as in the Sieverts static gas case shown in Figure 2c), but there is the strongest variation of all the gas flow measurements for CO_2_/H_2_ dual gas flow: (0.048 ± 0.002) nm. By comparison, the increases in the linker *d*-spacing for dual gas adsorption in the Sieverts static gas case (Figure 2a–c) are 0.056 nm, 0.057 nm and 0.065 nm, respectively, for CO_2_/N_2_, CO_2_/CH_4_ and CO_2_/H_2_, each with an estimated standard deviation uncertainty of ±0.015 nm.

We conclude that NiBpene is at least partially selective for CO_2_ adsorption/desorption in the case of a pure single gas, under both static and flow conditions. This is evidenced not only by the absence of significant changes in the linker *d*-spacing for gases other than CO_2_, but also by the complete absence of significant changes anywhere in the associated XRD pattern, as illustrated in the Supporting Information Figure S4. However, this selectivity probably does not extend significantly to the case of dual gas adsorption under static gas pressure or gas flow conditions due to likely cooperative gate opening effects.

### 3.3. Structural Changes in NiBpene During CO_2_ Adsorption/Desorption under Supercritical Conditions

**Figure 3:**
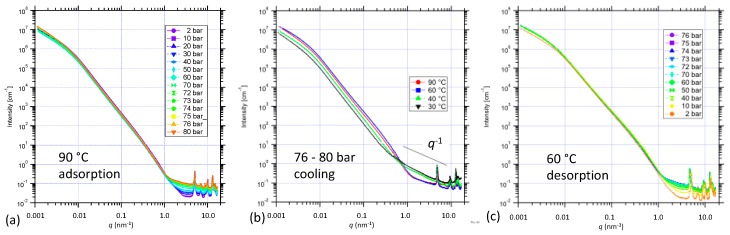
Combined USAXS/SAXS data for NiBpene during (**a**) adsorption of CO_2_ to 80 bar in the supercritical fluid regime; (**b**) cooling from 90 °C to 30 °C under 76 bar to 80 bar $p_{{\mathrm{CO}}_{2}}$; and (**c**) final desorption to 2 bar at 60 °C. Uncertainties are represented by the (small) scatter within each dataset.

New combined USAXS/SAXS/WAXS results were obtained for NiBpene under both subcritical (see Supporting Information Figures S5–S7) and supercritical CO_2_ static (no flow) pressure conditions during both adsorption and desorption, at controlled temperatures in the 60 °C to 90°C regime. For the case of increasing CO_2_ pressure at 90 °C from the sub-critical pressure regime up into the supercritical regime for CO_2_ pressures above ≈ 73 bar [69], Figure 3a presents combined USAXS and sector-averaged pinhole SAXS data on a log-log plot. There is a clear increase in the scattering background as CO_2_ is adsorbed and fills the pores space. The USAXS data are slit-smeared, which distorts the profile at low *q*, but model fits can allow for this, as described elsewhere, and can provide information on the NiBpene powder morphology [45]. While this is not the focus of our discussion here, we note that the data are absolute-intensity calibrated, and thus, intensity variations of the USAXS profile can be associated with the scattering contrast factor between the adsorbate gas and the solid NiBpene sorbent, and/or changes in the sample morphology. In this connection, Figure 3b presents corresponding USAXS/SAXS data for NiBpene subject to static CO_2_ pressures between 76 bar and 80 bar for progressively lower temperatures: 90 °C, 60 °C, 40 °C, and 30 °C. Two effects are apparent: (i) a pronounced increase in background under the XRD peaks; (ii) a significant decrease in intensity in the USAXS regime. Both effects are more pronounced as the transition from supercritical CO_2_ to liquid CO_2_ is approached and traversed for temperatures below 31 °C [67]. The changes are consistent with the presence of CO_2_ of progressively increased density both outside and within the NiBpene matrix: The dense CO_2_ both reduces the scattering contrast between the NiBpene solid and the surrounding gas environment and increases the X-ray scattering background. We also note the emergence of a *q*^−1^ scattering dependence extending from ≈0.5 nm^−1^ to ≈5 nm^−1^. This is the hallmark of extended linear scattering features that may be associated with our DFT predictions of changes in the Bpene ligand configuration as CO_2_ is adsorbed into the system in the supercritical and liquid CO_2_ regimes. Figure 3c shows corresponding data for the final desorption stage of these in situ measurements, which was conducted at 60 °C. We note very little change in the system, other than a decrease in the scattering background under the XRD peaks until the pressure is below 10 bar, which is consistent with the hysteresis in CO_2_ adsorption and desorption observed in the sub-critical regime.

**Figure 4:**
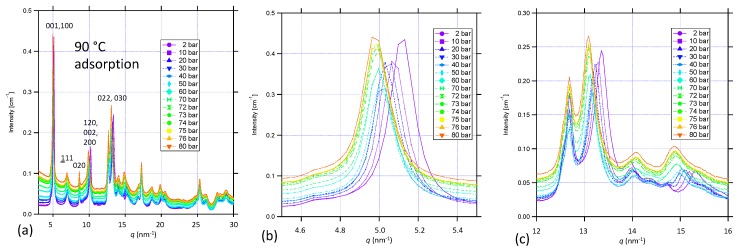
Combined SAXS/WAXS XRD patterns for (**a**) NiBpene during CO_2_ adsorption to 80 bar in the supercritical fluid regime; (**b**) expanded plot of Bpene linker XRD peak at *q* ≈ 5 nm^−1^; and (**c**) expanded plot of XRD peak at *q* ≈ 13 nm^−1^. Uncertainties represented by (small) scatter within each dataset.

**Figure 5:**
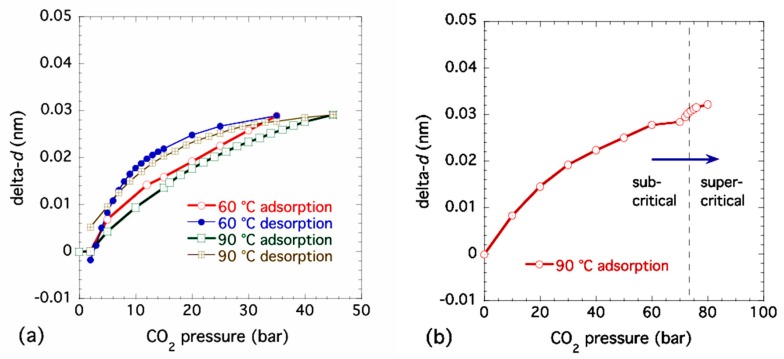
Changes in Bpene linker *d*-spacing with CO_2_ pressure for (**a**) adsorption and desorption at 60 °C and 90 °C under subcritical conditions and (**b**) adsorption only for pressures extending to 80 bar in the supercritical CO_2_ regime at 90 °C. Peak fit uncertainties are smaller than the data points.

Figure 4a presents combined SAXS and WAXS data showing the XRD peaks on a linear–linear plot corresponding to the same sequence of increasing CO_2_ pressures at 90 °C as Figure 3a. Based on previous work and our unit cell interpretation employed here, some major peak assignments are also included. Figure 4b,c presents expanded plots of the Bpene linker (001) peak at *q* ≈ 5 nm^−1^ and the (221) XRD peak complex at *q* ≈ 13 nm^−1^. Clearly, several of the XRD peaks including the Bpene linker spacing XRD peak move to the left (smaller *q*, larger *d*-spacing) as the CO_2_ pressure is increased. (This contrasts with the case shown in the Supporting Information Figure S4). However, some XRD peaks show no change during adsorption or desorption. Changes in the (001) XRD peak linker *d*-spacing have been determined through Lorentzian peak fitting. Examples of this XRD peak fitting are given in the Supporting Information Figure S8. The changes in lattice spacing, delta-*d*, from the ambient (1-bar) *d*-value are plotted against CO_2_ pressure for new sub-critical adsorption and desorption measurements at both 60 °C and 90 °C in Figure 5a, where the increase in linker *d*-spacing on adsorption at these temperatures is (0.029 ± 0.001) nm. For a separate sample run, changes in linker *d*-spacing are shown in adsorption only with pressures increasing into the supercritical regime at 90 °C in Figure 5b. There is no corresponding desorption data at 90 °C as the final desorption was conducted at 60 °C. Allowing for the use of different powder samples and temperatures and inevitable small differences in starting conditions, these results are consistent with those presented earlier in Figure 2. We also note from Figure 4b and Figure 5b that the Bpene linker *d*-spacing undergoes a further expansion of ≈ 0.003 nm once $p_{{\mathrm{CO}}_{2}}$ is increased from 72 bar into the supercritical regime to 80 bar. This point is discussed in regard to DFT predictions of CO_2_ adsorption in NiBpene in Section 3.5.

### 3.4. Lattice Parameter Determination of NiBpene under Selected CO_2_ Pressure Conditions

**Figure 6:**
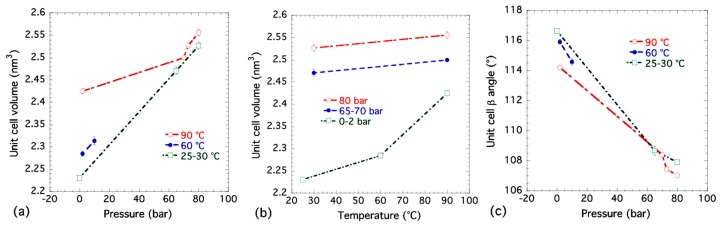
Variation of NiBpene unit cell parameters with CO_2_ pressure and temperature: (**a**) unit cell volume, V, versus CO_2_ pressure, $p_{{\mathrm{CO}}_{2}}$; (**b**) unit cell volume, V, versus temperature, T; and (**c**) unit cell *β* angle versus $p_{{\mathrm{CO}}_{2}}$. Vertical bars indicate standard deviation uncertainties for each data point.

Table 1 gives results for least-squares refinements of eight selected XRD pattern datasets collected from the combined USAXS/SAXS/WAXS measurements. For datasets 1 to 4, datasets 5 and 6, and datasets 7 and 8, cell parameters are compared as a function of $p_{{\mathrm{CO}}_{2}}$ at fixed temperatures of 90 °C, 60 °C and 30 °C, respectively. Dataset 9 was measured at room temperature under ambient pressure. Figure 6 shows how the unit cell volume and *β* angle vary with pressure ($p_{{\mathrm{CO}}_{2}}$) and temperature (T). The general trends shown in the unit cell volume plots are that, as pressure is increased, the unit cell volume, *V*, also increases due to the greater number of CO_2_ molecules residing inside the unit cell. At the same time, the unit cell *β* angle decreases and moves closer to 90° indicating that the Bpene ligand is in a more perpendicular and upright orientation. Meanwhile, as temperature is increased, the unit cell volume generally expands in accordance with expected thermal expansion.

It appears that at the supercritical pressure (starting at 73 bar, 90 °C), the *β* angle starts to decrease more strongly with increased pressure while the unit cell volume increases more steeply. Among all of the eight selected XRD measurements, the *β* angle is smallest at the highest supercritical pressure used at 80 bar, even though it is still larger than the 97° found in the previous single crystal study where DMSO was the sorbate [44]. It is conceivable that the Bpene ligands interact with the DMSO molecules to open up the cavities within the NiBpene structure to a greater degree than occurs due to the packing of more polarizable CO_2_ molecules. The CO_2_ molecules have to align themselves to maximize their packing density within the structure, as discussed in connection with the DFT results. Even with supercritical CO_2_ pressures, the unit cell does not attain as high a volume as that found in the single crystal study with DMSO as the sorbate and with an extra Bpene ligand. This may simply be due to larger molecular cross sections for DMSO and Bpene guests compared to CO_2_.

### 3.5. DFT Model Results for NiBpene

Individual Bpene molecules prefer to orient themselves such that the projected molecule in the *bc* plane extends along either the *b* or *c* direction (Figure 1). The barrier for 90° rotation of a single Bpene molecule is estimated to be about 0.35 eV. Four types of favorable collective ordering were found (Figure 1): (1) where all Bpene molecules have their projection along the *b* axis ([b] type); (2) where the projected orientations alternate along *b* and *c* ([bc] type), (3) [c] type, and (4) canted [c] type (Figure 1c) where the Bpene molecules rotate slightly to avoid interfering with their neighbors along the *c* direction. Note that the canted [c] type describes the orientation pattern of the bound Bpene molecules in the structure refinement of Wong-Ng et al. [44]. Note also that the Bpene molecule lacks 180° rotational symmetry around its long axis and thus, each Bpene in each structure shown in Figure 1 actually has two possible non-equivalent orientations. Here, we present results for the specific orientation patterns found with *P2/c* monoclinic symmetry and lowest energy.

**Figure 7:**
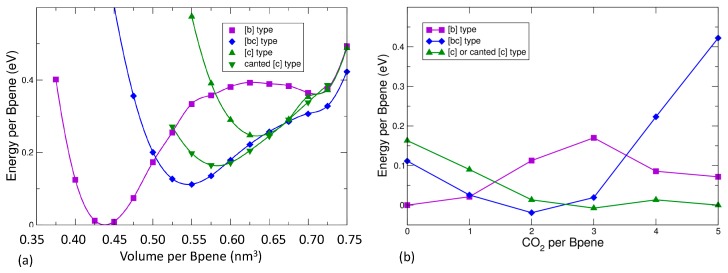
(**a**) Predicted DFT energy versus volume for empty NiBpene for various Bpene orientational patterns; (**b**) predicted DFT relative energy per Bpene for different types of Bpene orientational patterns and different CO_2_ loadings. The effective chemical potential is set so that the minimum energy is zero for 0 and 5 CO_2_ per Bpene.

The experimental evidence is consistent with higher CO_2_ pressure leading to higher CO_2_ sorption leading to expansion of the unit cell volume and a decrease of the monoclinic *β* angle towards 90°. Prior to presenting the DFT results for CO_2_ sorption, Figure 7a gives the results of energy versus volume for an empty NiBpene structure. For completely empty NiBpene, DFT actually predicts a collapse of the structure to a volume of less than 0.45 nm^3^ per Bpene. As the volume increases, three transitions occur, to a [bc] type, then a canted [c] type, and finally back to a [bc] type. Note that these types of transitions may appear to be first-order; however, one needs other types of measurements (such as heat capability measurements) to corroborate any such conclusion. The predicted series of orientational ordering phase transitions is different when CO_2_ molecules are incorporated into the DFT calculations (Figure 7b). Now, a transition from a [b] type, to a [bc] type, to a canted [c] type, is predicted as the CO_2_ loading increases. No transition back to a [bc] type occurs because this orientational pattern (Figure 1b) has less empty volume for the CO_2_ molecules to occupy than the other types.

**Figure 8:**
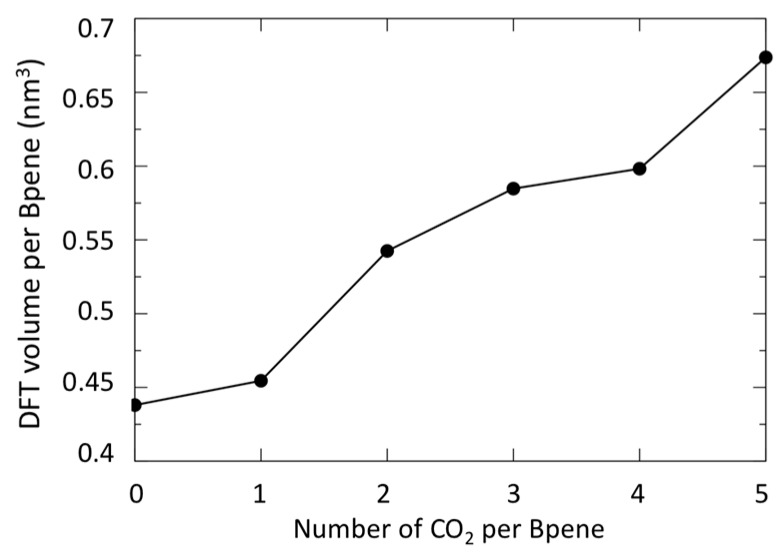
DFT predicted cell volume versus CO_2_ loading.

**Figure 9:**
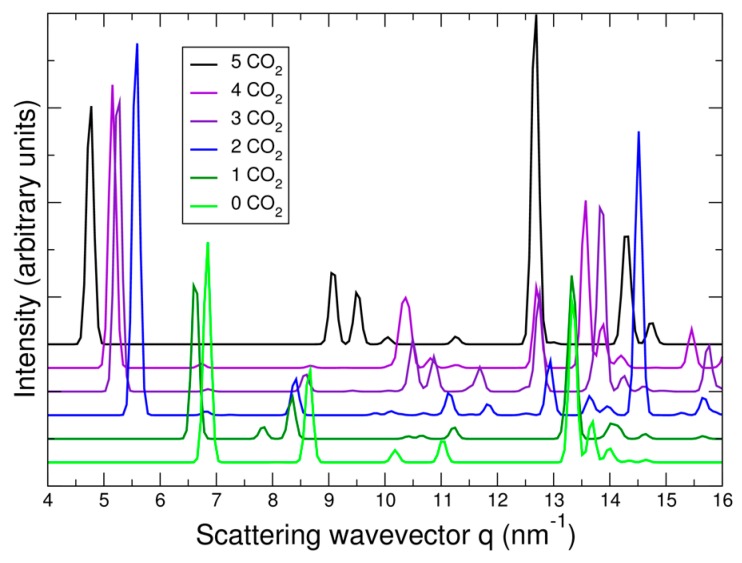
X-ray powder diffraction patterns of minimum energy DFT structures in NiBpene versus carbon loading.

In Figure 8, the predicted cell volume versus CO_2_ loading is shown. Note that zero-point and thermal motion of the Bpene atoms and the CO_2_ are not included. These effects are expected to expand the volumes above the DFT predictions. In Figure 9, the simulated powder XRD patterns as a function of CO_2_ loading are shown based on the lowest-energy DFT structure at each CO_2_ loading. Atomic structure factors and Lorenz factors are included, but the unknown temperature factors are neglected. The simulated powder patterns show intriguing similarities to the experimental ones. The first strong peak is always the [100] peak, and the *q*-shifts of this peak inversely correlate with the distance between the Ni[CN]_4_ planes. A small peak near 6.8 nm^−1^ is sometimes visible and sometimes absent as is true experimentally. A set of specific diffraction peaks near this value are identified; these peaks have supercell [hkl] indices where k or l, or both, are odd, and will therefore be absent if the NiBpene structure has the translational symmetry of a 1.4 nm × 0.7 nm × 0.7 nm cell. It is interesting that the [bc] and canted [c] type Bpene arrangements of Figure 1 inherently lead to some supercell periodicity. However, more work is needed to make a quantitative agreement between the experimentally measured powder XRD patterns and structural models.

**Figure 10:**
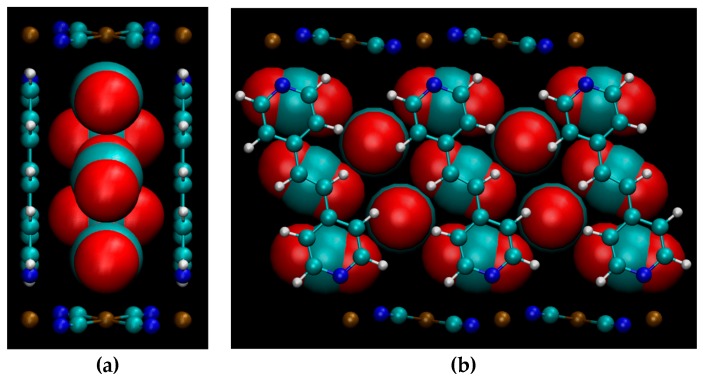
Predicted geometry of CO_2_ at loading of five CO_2_ per Bpene, projected along (**a**) the *ab* plane and (**b**) the *ca* plane. CO_2_ molecules are magnified for clarity. Element key: gold = Ni, white = H, blue = N, green = C, red = O. Also see Figure 1.

The calculated geometry of NiBpene loaded with 5 CO_2_ per Bpene is shown in Figure 10. It contains a packing of three CO_2_ aligned mostly along the *c* direction alternating with two CO_2_ aligned mostly along the *b* direction. DFT calculations show that it is energetically unfavorable for NiBpene to accommodate a sixth CO_2_ molecule per Bpene. These results compare with a maximum adsorption between 3 and 3.5 CO_2_ per Bpene, measured previously for sub-critical CO_2_ isotherms measured at 30 °C (Figure 2).

A thermodynamic analysis via DFT or classical modeling to fully bridge the DFT results with the experimental measurements would require advances in modeling far beyond the scope of this work. Note that existing thermodynamic modeling, e.g. Reference [70], generally focuses on systems with a “rigid” framework and that modeling of flexible MOFs is more difficult. Thermodynamic modeling of one flexible MOF system, MIL-53, has been successfully performed [71], but the Bpene orientational degrees of freedom make Ni-Bpene much more difficult to model, either here or in any future work. Here, the focus of our DFT studies has been to explore the coupling between the amount of CO_2_ adsorption, cell size, and Bpene orientational configuration in detail.

Adsorption of a single ad-molecule per Bpene for different molecules was investigated using a canted [c] type configuration with a volume of 0.575 nm^3^ per Bpene. CO_2_, N_2_, CH_4_, and H_2_ were all found to favor a position near the exposed Ni site. The calculated adsorption energies per molecule were 0.37 eV, 0.25 eV, 0.36 eV, and 0.13 eV, respectively. The DFT results, in combination with the XRD results suggest that CO_2_ sorption hysteresis are due to transitions between different ordering types of the Bpene molecules as the pressure changes, in line with the picture of Culp et al. [23]. The different ordering types have different periodic cells, leading to the appearance or disappearance of diffraction peaks. The effects observed for supercritical CO_2_ may be due to a transition from a canted [c] configuration to a [c] configuration concomitant with an increase of CO_2_ sorption to 5.0 per Bpene.

## 4. Concluding Discussion

In this paper, we have discussed the structural basis of CO_2_ adsorption in the MOF material: Ni(1,2-bis(4-pyridyl)ethylene)[Ni(CN)_4_] (NiBpene), a representative model flexible MOF exhibiting a CO_2_ sorption isotherm with characteristic hysteresis and features that can be associated with discrete structural changes. While our results have demonstrated a structural response in the case of pure CO_2_ adsorption and desorption, there is little or no change in the structure in the presence of pure N_2_, CH_4_ or H_2_ under static and flowing gas pressure conditions (at least over the pressure ranges tested), selective gas adsorption for CO_2_ with respect to N_2_, CH_4_ and H_2_ under dual gas static and flowing gas pressure conditions cannot be established. Further experimental work and DFT calculations are needed to determine a full explanation of why this is so. We have highlighted the importance of solving the MOF crystal structure, even if this varies for different guest species. This is because knowledge of the structural arrangement allows DFT to work with the near-neighbor bonds and force constants to make predictions regarding the structural response of the material during adsorption and desorption of guest species, and hence, to explain the observed structural transitions. Due to the hybrid nature of flexible MOFs, such predictions can be exploited to tune the overall host–guest interactions [72,73,74,75]. We note that DFT predicts different calculated adsorption energies per molecule for CO_2_ with respect to either N_2_ or H_2_, and this may underlie some preference of NiBpene for pure CO_2_ adsorption over these other two gases when CO_2_ is not present. The preference for pure CO_2_ adsorption over pure CH_4_ adsorption is less clear on this basis. However, it may originate, at least in part, from the different shape and size of the CH_4_ molecule with respect to CO_2_.

Using in situ operando small-angle neutron and X-ray scattering, neutron diffraction and X-ray diffraction, we have compared the structural effects of both single gas and dual gas adsorption and desorption at pressure, under both static (Sieverts) and gas flow conditions. We find these effects, including the possible cooperative gate-opening of one gas to allow another to adsorb, broadly consistent between static and flowing gas conditions. However, we note that single and dual gas flow conditions, with an effectively unlimited supply of the component gases, are much more likely than static Sieverts gas conditions to approximate the conditions under which these flexible MOF materials will be called to function.

Finally, we note the enhanced CO_2_ adsorption that appears to occur in NiBpene for gas pressures sufficiently high to put CO_2_ into the supercritical regime. This is manifest both in the enhanced expansion of the (001) linker *d*-spacing under these conditions and in the DFT calculations that suggest how the number of CO_2_ molecules per Bpene can be increased from the previously observed maximum of ≈4 to ≈5 CO_2_ molecules per Bpene. The potential efficiency of supercritical CO_2_ for solvent applications suggests that the exploitation of CO_2_ adsorption in the supercritical regime may provide an important role for such flexible MOF materials in the future.

## Figures and Tables

**Table 1:** Estimated unit cell parameters of 9 XRD datasets (sets 1–8 from USAXS/SAXS/WAXS measurements and set 9 from powder XRD data taken at room temperature and ambient pressure.).

Dataset	T (°C)	$p_{{\mathrm{CO}}_{2}}$ (bar)	*a* (nm)	*b* (nm)	*c* (nm)	*β* (°)	*V* (nm^3^)
1	90	80	1.373(2) ^1^	1.447(4)	1.3513(9)	107.06(9)	2.556(8)
2	90	73	1.3647(9)	1.4353(7)	1.3522(15)	107.45(10)	2.527(4)
3	90	70	1.3683(15)	1.4301(6)	1.3469(9)	108.43(7)	2.500(3)
4	90	2	1.3597(6)	1.442(2)	1.356(3)	114.22(6)	2.425(6)
5	60	10	1.357(4)	1.4087(12)	1.332(3)	114.6(2)	2.314(9)
6	60	2	1.360(3)	1.4080(9)	1.327(2)	115.92(15)	2.285(6)
7	30	80	1.3616(9)	1.452(4)	1.344(2)	107.92(9)	2.527(8)
8	30	65	1.357(3)	1.437(2)	1.338(3)	108.7(3)	2.471(8)
9	25	0	1.3529(1)	1.3966(2)	1.321(2)	116.66(6)	2.231(4)

^1^ Standard deviation uncertainties in least significant digits given in parentheses.

## Supplementary Materials

The following are available online at https://www.mdpi.com/2079-4991/9/3/354/s1, Figure S1: Schematic of USAXS/SAXS/WAXS measurement configurations with dual gas flow system; Figure S2: Schematic of USAXS/SAXS/WAXS measurement configuration with capillary sample cell and syringe pump; Figure S3: SANS diffraction peak fits for single and dual gas static conditions; Figure S4: Combined SAXS/WAXS XRD data for in situ pure H_2_ gas flow; Figure S5: Combined USAXS/SAXS data for in situ subcritical CO_2_ adsorption/desorption; Figure S6: Combined SAXS/WAXS XRD data for in situ subcritical CO_2_ adsorption/desorption; Figure S7: Detailed XRD data for combined (001)/(100) peak; Figure S8: Examples of XRD Lorentzian peak fits in SAXS/WAXS data. Also available is an electronic zip file containing: (i) Files in *cif format giving the minimum DFT energy structures for each CO_2_ loading factor from 0 to 5 in Ni-Bpene; (ii) Files in VASP POSCAR format giving the minimum DFT energy structures for each CO_2_ loading factor from 0 to 5 in Ni-Bpene; (iii) The VASP KPOINTS file used; (iv) A sample VASP INCAR file used for the relaxations; (v) Titles of the VASP pseudopotentials used; and (vi) A README file that links these Supplementary Information files to the manuscript and describes what each file contains.
