# Supplementary material for: Structural Basis of CO2 Adsorption in a Flexible Metal-Organic Framework Material
Source: Nanomaterials (Basel). 2019 Mar 4;9(3):354. doi: 10.3390/nano9030354 (PMC6473970; doi:10.3390/nano9030354)
Supplement: Supplementary file 1 [file nanomaterials-09-00354-s001.zip › nanomaterials-449810-supplementary-final/nanomaterials-449810-supplementary-final.pdf]

nanomaterials

Supplementary Material

Structural basis of CO_2 adsorption in a flexible metal-organic framework material

Andrew J. Allen [1,*], Winnie Wong-Ng [1], Eric Cockayne [1], Jeffrey T. Culp [2,3] and Christopher Matranga [3]

[1] Material Measurement Laboratory, National Institute of Standards and Technology (NIST), Gaithersburg, MD 20899-8520, USA; winnie.wong-ng@nist.gov (W.W.-N); eric.cockayne@nist.gov (E.C.)
[2] AECOM Corporation, Pittsburgh, PA 15236, USA; jeffrey.culp@contr.netl.doe.gov
[3] National Energy Technology Laboratory (NETL), US Department of Energy, Pittsburgh, PA 15236, USA; matranga@netl.doe.gov
* Correspondence: andrew.allen@nist.gov; Tel.: +1-301-975-5982

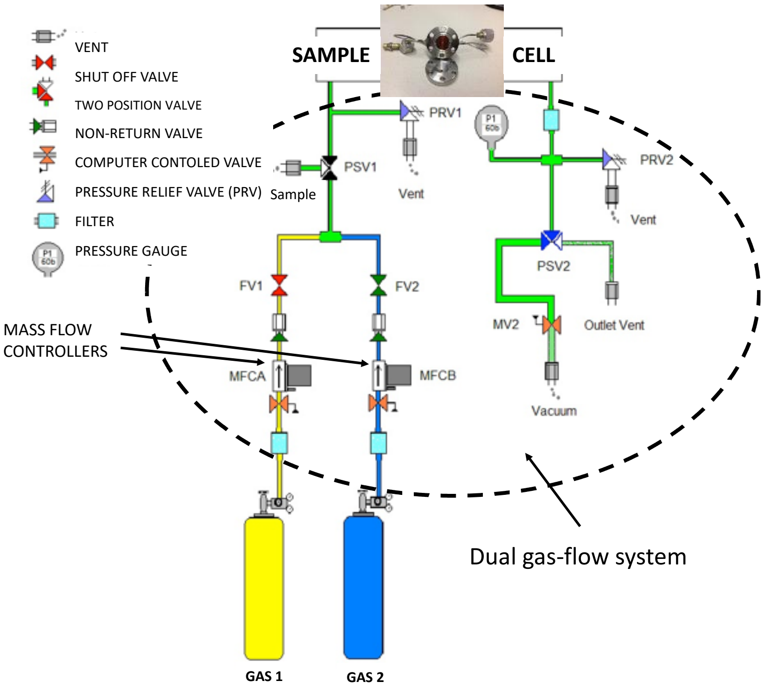

Figure S1. Schematic of USAXS/SAXS/WAXS measurement configurations using automated dual gas flow system. One or two gases can be used with total pressures up to 50 bar and flow rates shared between the 2 gases. Adapted from [1] with permission from the International Union of Crystallography (IUCr), 2018.

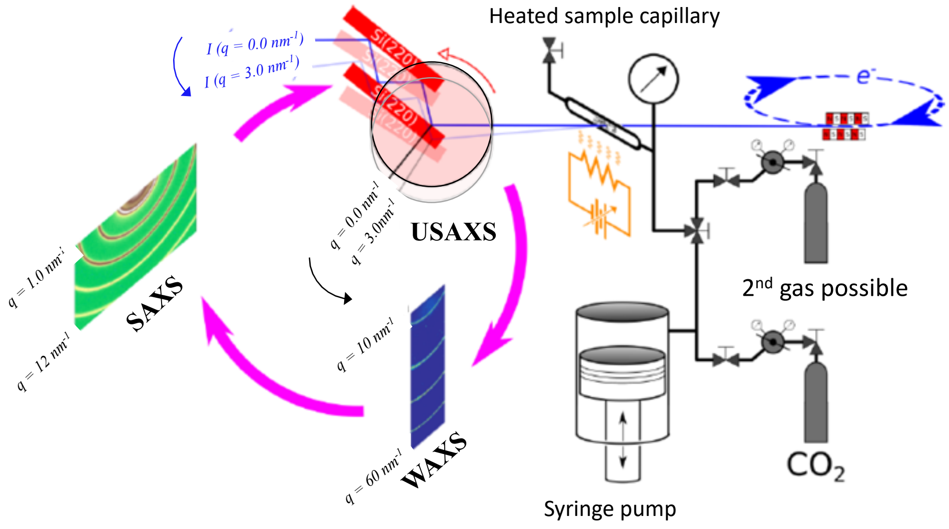

Figure S2. Schematic of USAXS/SAXS/WAXS measurement configuration with heated capillary sample cell and syringe pump system for CO_2 pressures up to 80 bar (supercritical). Adapted from [1] with permission from the International Union of Crystallography (IUCr), 2018.

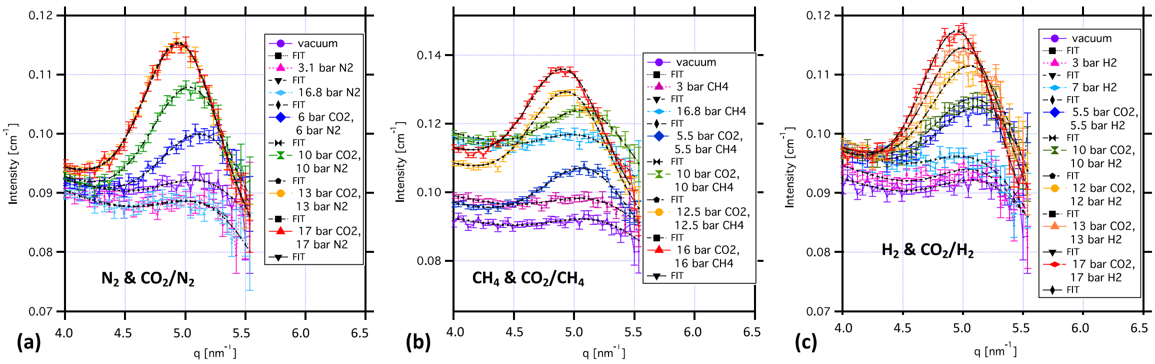

Figure S3. SANS diffraction peak fits for single and dual gas static conditions: **(a)** N_2 and CO_2/N_2; **(b)** CH_4 and CO_2/CH_4; **(c)** H_2 and CO_2/H_2. These results follow on from measurements reported in [2]. Vertical bars are statistical standard deviation uncertainties for each data point.

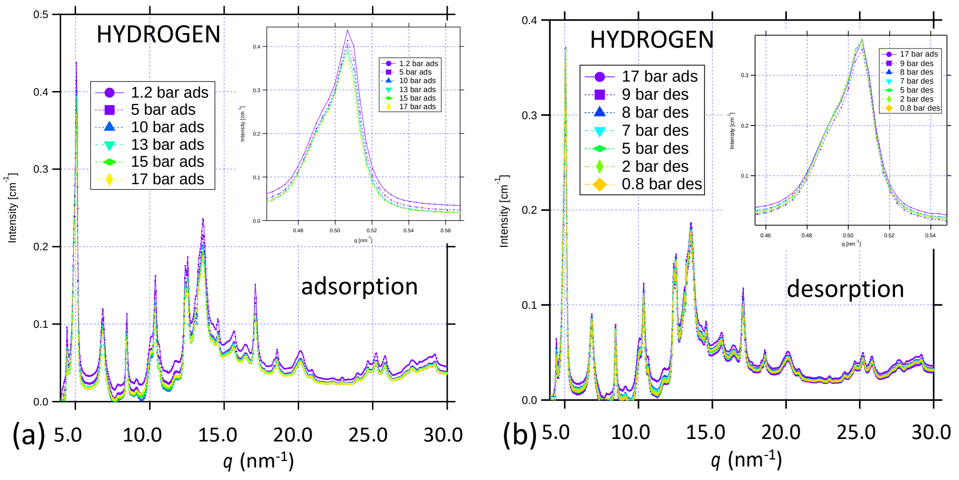

Figure S4. Combined SAXS/WAXS XRD data for *in situ* pure H_2 gas flow: **(a)** adsorption and **(b)** desorption at ≈ 30 °C. Uncertainties given by scatter in data. No changes in XRD peak position are

apparent. Insets are for linker *d*-spacing peak. Small peak at lowest *q* is an artifact of the polyamide pressure cell windows.

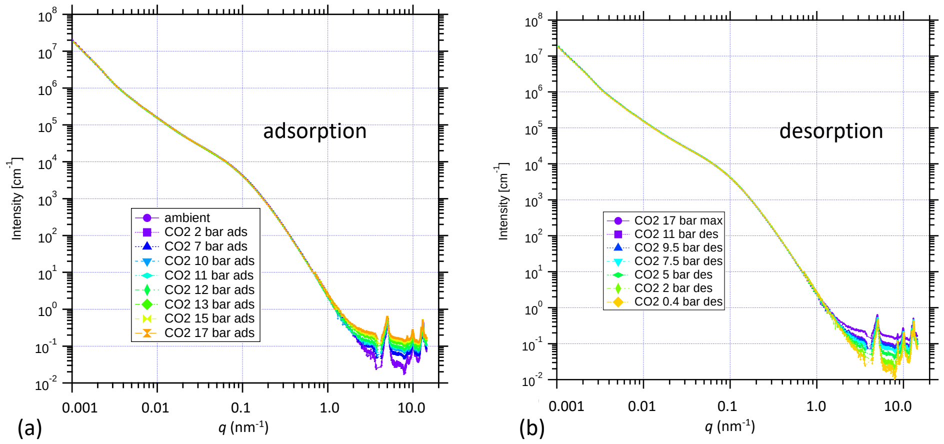

Figure S5. Combined USAXS/SAXS data for *in situ* subcritical CO_2 **(a)** adsorption and **(b)** desorption using capillary gas cell arrangement at 90 °C. Uncertainties are given by (small) scatter in the data.

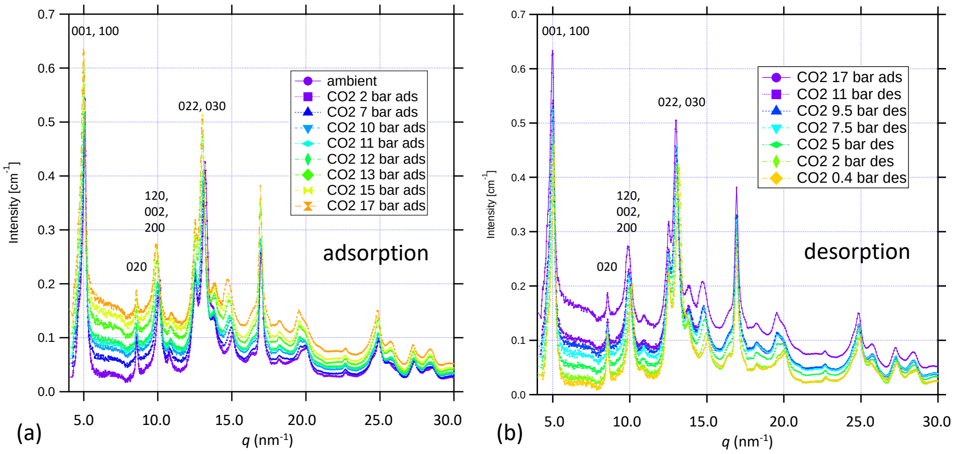

Figure S6. Combined SAXS/WAXS XRD data for *in situ* subcritical CO_2 **(a)** adsorption and **(b)** desorption using capillary gas cell arrangement at 90 °C. Uncertainties given by scatter in data.

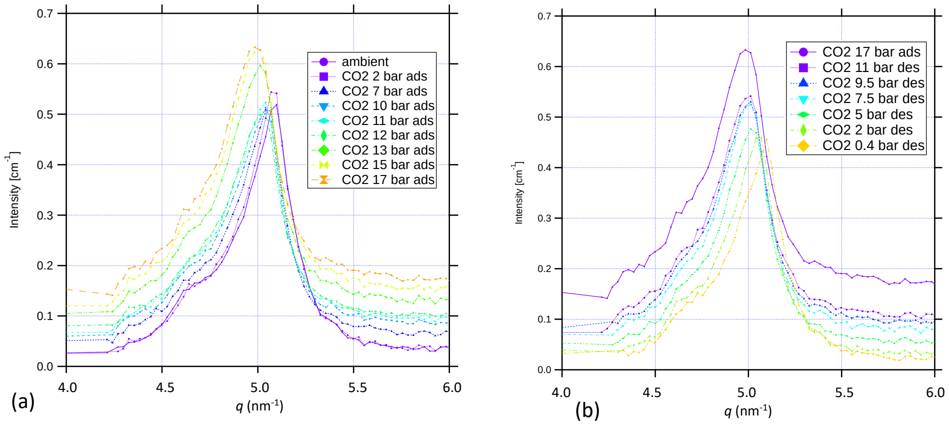

Figure S7. Detailed XRD data for combined (001)/(100) peak during *in situ* subcritical CO_2 **(a)** adsorption and **(b)** desorption using capillary flow cell arrangement at 90 °C. Uncertainties given by scatter in data.

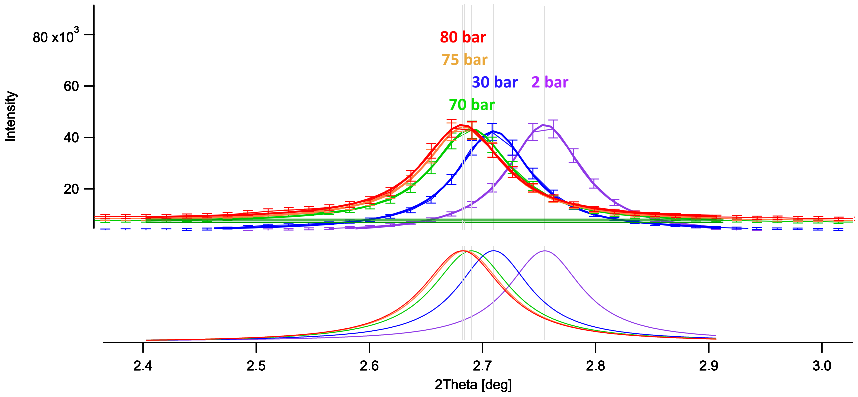

Figure S8. Examples of XRD Lorentzian peak fits in SAXS/WAXS data plotted as intensity versus scattering angle, 2Theta, for CO_2 adsorption extending into supercritical regime at 90 °C. Vertical bars at each data point are standard deviation uncertainties. The fitted Lorentzian peak functions are reprised below the data plots, along the displaced horizontal axis showing peak diffraction angle, in order to clarify small changes in peak position. See [3] for summary of analysis method.

References

1. Ilavsky, J.; Zhang, F.; Andrews, R.N.; Kuzmenko, I.; Jemian, P.R.; Levine, L.E.; Allen, A.J. Development of combined microstructure and structure characterization facility for in situ and operando studies at the Advanced Photon Source. *J. Appl. Cryst.* **2018**, *51*, 867–882, doi:10.1107/S160057671800643X.

2. Allen, A.J.; Espinal, L.; Wong-Ng, W.; Queen, W.L.; Brown, C.M.; Kline, S.R.; Kauffman, K.L.; Culp, J.T.; Matranga, C. Flexible metal-organic framework compounds: In situ studies for selective CO_2 capture. *J. Alloy. Compd.* **2015**, *647*, 24–34, doi:10.1016/j.jallcom.2015.05.148.

3. Ilavsky, J.; Jemian, P.R. *Irena*: Tool suite for modeling and analysis of small-angle scattering. *J. Appl. Cryst.* **2009**, *42*, 347–353, doi:10.1107/S0021889809002222.

© 2019 by the authors. Submitted for possible open access publication under the terms and conditions of the Creative Commons Attribution (CC BY) license (http://creativecommons.org/licenses/by/4.0/).
